# Some Remarks on Fistula in Ano

**Published:** 1898-03

**Authors:** William S. Goldsmith

**Affiliations:** Atlanta, Ga.; Lecturer on Genito-Urinary and Rectal Diseases, Atlanta Medical College


### SOME REMARKS ON FISTULA IN ANO.

By WILLIAM S. GOLDSMITH, M.D.,
Lecturer on Genito-Urinary and Rectal Diseases, Atlanta Medical College.

In a paper entitled "Fistula in Ano," read before the State Society last April, I endeavored to briefly point out what I considered the principles of treatment of this affection. Since that time I have had under observation a case which presented certain conditions that cannot fail to be of interest, as it involves a question of some surgical importance. But, before further reference is made to this case, I desire to comment on the various divisions and nomenclature employed by authors of text-books on rectal diseases, and, indeed, all contributors on this subject in text-books on general surgery. These divisions are misleading to a medical student, and no doubt also to the practitioner of medicine.

I have, in my humble capacity as a teacher, explained to students the erroneous varieties of so-called fistula in ano. A fistula, or as the text-books describe, a complete fistula, and a sinus, are used synonymously, the difference being expressed by a qualifying adjective. A good definition of the two terms is found in Park's Surgery, Vol. 1, which is as follows: "These are terms applied to more or less tubular channels abnormally connecting various parts of the body, or connecting some cavity with the surface of the body in a way anatomically quite abnormal. . . . . A more exact distinction between the two terms would imply that a sinus connects the surface with some deeper portion where a cavity is not normally present, *i. e.*, with a focus of disease; whereas a fistula properly refers to a tubular passage connecting natural or pre-existing cavities in an abnormal manner."

There is no mistaking such a lucid description, yet we have complete, blind internal, blind external, and horseshoe fistulas. Blind internal fistulas are a rare occurrence, or rather it is a condition not usually suspected until other grave complications arise. The so-called blind external fistulas are quite frequently seen, and the horseshoe variety, absurdly named, is usually a complication of a fistula and numerous sinuses. I hold that there is but one variety of fistula in ano, and that is the complete. This latter is well named, for to be a fistula it must be, of course, complete. Anything short of that condition would necessarily define it as a sinus.

Blind external fistulas (for the moment retaining the name) are the result of an abscess, either superficial or ischio-rectal. As the name implies it has no connection with the rectum, therefore it cannot be a fistula, but is a sinus. This leads me to the case in mind. In December, 1896, the gentleman affected had an ischio-rectal abscess, which pointed about two inches from the anus. After poulticing the part for a day or two, the attending physician opened the abscess, evacuating a quantity of pus, and found that there was a cavity which extended toward the rectum and out into the buttock. This cavity was at once cleansed with peroxide of hydrogen, and irrigated daily with a bichloride of mercury solution. The wound was drained by means of a gauze tent, and for a while there was some evidence of repair. I was called in consultation the latter part of March and concurred in the opinion that an operation, under ether, was necessary. This was done, and the entire lining membrane was curetted and dissected out, supplemented by the free use of the peroxide of hydrogen spray. Careful search failed to reveal the least indication of a tract running up alongside the rectum, other than the superior boundary of the abscess cavity, which had, in its ramifications, destroyed tissue along the rectal wall for perhaps an inch and one-half. The wound healed kindly, and led us to believe that a certain cure would result. A month after the operation I discovered a suspicious exudation of pus, and the probe confirmed my fears. A tract an inch and one-half in depth was found, following the inner boundary of the old abscess cavity, and pointing near the rectum. All efforts to heal this sinus proved futile. I advised another operation, which was deferred from time to time until the first of October.

Upon again opening this tract I found that, owing to its tortuous course, only one-half of its depth was accessible to the probe, and that it ran up the rectal wall for three inches. I searched for an internal opening and was not disappointed at my failure to find one. This circumstance does not, of course, deter us from converting a sinus into a fistula as the preliminary technique in performing the radical operation. I then incised the gut with all intervening tissue, including the sphincters. The last operation resulted in complete cure. This is the point I wish to make, are we justified in cutting the gut and the sphincter muscles, incurring the risk of imperfect union, in abscesses, where the cavity extends to the rectal wall and above the sphincters? In this case I did not feel at that time, that I should undertake such a procedure, but I have made up my mind that should a similar case come to me, I would have no hesitation in doing the radical operation, that is making a complete section of all intervening structures.

In deep rectal abscesses I think it is our duty to inform the patient of the likelihood of a subsequent fistula, and advise the administration of an anesthetic for the apparently simple operation of opening the abscess. When this is done we are free to act as our judgment dictates, and have abundant opportunity of laying open the entire circumscribed area, together with the almost absolute certainty of successfully removing all morbid tissues. All abscesses are painful, and as we all know, every patient having a rectal ailment, looks upon the invasion of that particular territory, with much trepidation. To attempt the thorough opening of a rectal abscess without an anesthetic is most unsatisfactory, and, at best, the assistance of cocain, is often immaterial, as nothing more than the mere skin incision is permitted. I am extremely cautious in the use of cocain about the rectum, and very seldom resort to it.

If, after the eradication of the lining membrane, an angle of the wound is directed to the gut, say above the internal sphincter, should we proceed to make an opening and incise all the tissues? On one hand we have the local and constitutional evidence of a violent inflammation, and on the other, the fact of our patient already under the influence of the anesthetic, and also the improbability of a successful issue, owing, among other things, to the peculiar anatomical construction of the parts.

My convictions are that under these circumstances we could conscientiously incise the gut, and proceed with the treatment, indicated for a true fistula in ano. In closing, permit me to make slight reference to the most pronounced local idiosyncrasy to iodoform I have ever seen. This occurred in this case. I seldom use iodoform gauze, and more rarely the powder, as a surgical dressing, except in rectal work, and as a medium of drainage. In this case, as is my custom, I packed the wound tightly for the first forty-eight hours with iodoform gauze. I pack these wounds tightly to stop what is sometimes a profuse oozing, and leave this dressing in place for two days on account of the fact that to remove it in a shorter time subjects the patient to much pain and a recurrence of the oozing. When I dressed the wound two days after the operation I found that wherever the iodoform gauze pad touched the skin, and even in a much larger area exposed to the drainage and blood-stains, a most violent dermatitis was excited. The irritation was so intense that nothing short of liberal hypodermics of morphine afforded relief. This experience should have been sufficient warning, but about two weeks later I inserted a pledget of iodoform gauze, the size of a lead pencil and two inches in length, into the tract, for the double purpose of drainage and of mitigating the unpleasant odor of the discharge. I was called in the middle of the night to give my patient some relief from the most torturing irritation and pruritus, the result of my indiscretion. I was told that the idiosyncrasy began to assert its peculiarity within an hour, but the observance of my directions prevented any action until all patience and forbearance was exhausted. I at once resumed the use of sterilized gauze, which answered my purpose in a thoroughly satisfactory manner. Bichloride gauze was also used sparingly, but having seen skin irritation arise from its use, I abandoned this dressing also.

*27–28 Grant Building.*

---

When you advise a patient to wear a suspensory bandage, tell him to get the kind which has straps running from the posterior band of the bag itself, around the legs or buttocks. The suspensories which are attached to the belt in front alone, with an elastic in the back edge of the bag, are useless.—*International Journal of Surgery.*

